# Model-based comparison of latency estimation methods for the pupillary light reflex

**DOI:** 10.1038/s41598-026-68921-9

**Published:** 2026-09-02

**Authors:** Marcel Schepelmann, Hans Georg Krojanski

**Affiliations:** Computational Health Informatics, Leibniz University Hannover, 30167 Hanover, Germany

**Keywords:** Pupillometry, Pupillary light reflex, Latency estimation, Synthetic data, Benchmarking, Dynamic pupil model, Engineering, Optics and photonics

## Abstract

Accurate estimation of pupillary light reflex (PLR) latency is important in clinical and research settings, yet reported results are often difficult to compare due to differences in hardware, sampling rates, and analysis methods. This study introduces a systematic and reproducible benchmark for evaluating PLR latency estimation algorithms using synthetic data with known ground-truth latency. Synthetic pupillograms were generated using an established dynamic model of the PLR, extended to handle short light impulses. Five commonly used latency estimation methods were evaluated under these conditions, with 1000 traces simulated per configuration. Performance was assessed using the mean absolute error (MAE) between estimated and ground-truth latency. Across many conditions, a method developed by Bergamin and Kardon, combining filtering, interpolation, and analysis of the first and second derivatives achieved promising results, although its performance deteriorated under high noise and low-intensity stimuli. In contrast, a simple piecewise linear fit showed consistent, moderate performance across configurations. Threshold-based detection performed well for strong and medium stimuli but degraded for weak responses, while derivative-based and exponential curve-fitting approaches showed higher sensitivity to noise or stimulus conditions. For practical use, we recommend the Bergamin and Kardon method as the default choice, whereas the piecewise linear fit may be preferable when the hardware or measurement conditions are unknown, such as in cross-device smartphone applications.

## Introduction

The PLR is the autonomic response of the pupil to light. Following an exposure to a light stimulus, the pupil constricts and gradually re-dilates once the stimulus is removed^1^. Pupillometry, the quantitative measurement of this response, is increasingly used in clinical diagnostics, neurophysiological research, and mobile health applications^2–5^. Among the measurable PLR features, latency—the delay between stimulus activation and the constriction onset–is of particular importance. Pathologic latency has been associated with outer retinal dysfunction and afferent damage^6,7^. Latency of the PLR was also reported as biomarker for multiple sclerosis^8,9^, Leber hereditary optic neuropathy^10^, amblyopia^11,12^, diabetes^13^ and concussions^2,4^.

Despite its significance, reliable latency estimation remains a nontrivial challenge^6,7^. Several factors contribute to this difficulty. First, commercially available pupillometers or laboratory prototypes operate at varying sampling rates ranging from 5 Hz to 2000 Hz^14–16^, determining the temporal precision with which small deflections in pupil diameter can be detected. Second, detection-based noise introduces variability that may obscure the early slope of the constriction onset. Third, physiological oscillations such as hippus can impose slow drift on the diameter signal^17–19^. These effects combine to produce uncertainty in the marking of the true latency, even under controlled experimental conditions. In a study by Capó-Aponte et al.^2^ involving 20 participants with concussions and a control group of 20 participants, a statistically significant difference was found between the latency of the two groups. However, the difference in the mean values of both groups was less than 30 ms. This further demonstrates that high accuracy is important. Furthermore, Bos states that a difference of over 20 ms for different estimation methods is unacceptable^15^.

A variety of methodological strategies have been proposed for latency estimation, including linear model fitting^20,21^, velocity deflections from zero^15^, maximal negative acceleration^6^ and curve-fitting using a second-order model^15^. However, comparing these methods objectively is challenging, as real PLR data does not provide an exact ground truth value for latency. Furthermore, experimental setups differ widely in illumination, device characteristics, and measurement noise. As a result, performance comparisons across studies remain inconsistent, and it is unclear how various latency estimation approaches behave under reduced frame rates, increased noise levels, or atypical physiological responses.

To address these issues, we propose a model-based framework that enables controlled comparison of latency estimation methods under reproducible conditions. By generating synthetic PLR signals with known ground-truth latency and configurable noise, drift, and sampling properties, the framework provides a systematic way to evaluate how different algorithms perform across a broad range of measurement scenarios. This approach not only introduces a new benchmarking but also reveals the conditions under which specific latency estimation strategies fail or remain robust.

## Related work

### Pupillary light reflex modeling

Mathematical models of the PLR can be categorized into equilibrium-state models and dynamic models^22^. Equilibrium-state models describe the steady-state relationship between pupil size and luminance and are typically derived from experimental measurements. Although they lack temporal dynamics, they are frequently incorporated into dynamic models^22^. Since latency estimation requires explicit temporal behavior, this review focuses on dynamic PLR models.

Longtin and Milton introduced one of the earliest dynamic PLR models based on a nonlinear delay differential equation^23^. Their model incorporates physiological latency by introducing a delayed retinal light signal and nonlinear iris mechanics, enabling the simulation of delayed and oscillatory pupil responses.

Usui and Hirata proposed a detailed biomechanical model of the human pupillary muscle plant, representing the sphincter and dilator muscles as viscoelastic elements^24^. While physiologically accurate, the resulting system of 19 differential equations is computationally expensive and difficult to parameterize, limiting its applicability for large-scale simulations^25^.

Pamplona and Oliveira combined the dynamic formulation of Longtin and Milton with the experimentally validated equilibrium pupil model of Moon and Spencer^26^, resulting in a hybrid model that captures both realistic steady-state behavior and temporal dynamics^27^. We follow this modeling strategy in our simulations; further details are provided in the subsection “Model implementation”.

To improve simulation accuracy for short light pulses, Fan and Yao proposed a second-order delayed differential equation model that reduces complexity compared to earlier approach from Usui and Hirata while retaining viscoelastic muscle behavior^25^. More recently, Zandi and Khanh combined the Fan and Yao model with a feed-forward neural network to enhance modeling flexibility^28^.

### Latency estimation in pupillometry

Early pupillometry studies reported substantial variability in PLR latency, but often failed to specify how latency was determined. For example, Ellis^1^ reported latencies ranging from 220 ms to 500 ms and observed a decrease in latency with increasing stimulus intensity; however, the method used to define response onset was not described.

Bos explicitly addressed this lack of methodological transparency and noted that only a minority of studies reported their latency detection approach^15^. He compared manual and automated latency estimation at different sampling rates and concluded that, at high sampling rates (*≈ *200 Hz), detecting deviations in pupil velocity from zero is sufficient, whereas at lower sampling rates (*≈ *50 Hz), curve-fitting approaches are more appropriate.

Bergamin and Kardon investigated the clinical relevance of PLR latency and highlighted the absence of a generally accepted estimation method^6^. They proposed an algorithm based on filtering, interpolation, and analysis of the first and second derivatives of the pupil signal. The method was developed using recordings from seven healthy subjects and evaluated on a larger cohort under the assumption that latencies in the left and right eyes are identical. The correlation coefficient between both eyes over several measurements was used as a metric to measure reliability. The authors further noted that increased spatial resolution may be more beneficial than very high sampling rates.

More recently, Jendritza et al.^7^ compared three different methods for determining PLR latency using pupillometry data from 150 healthy subjects. The evaluated approaches were based on linear fits to the baseline pupil diameter and the descending part of the pupillogram, using different numbers of data points. Test–retest reliability was used as the primary evaluation metric. Among the tested approaches, the method using a reduced fitting window during the pupillary constriction phase proved to be the most robust, particularly for small constriction amplitudes and noisy data.

Across these studies, latency estimation methods are typically evaluated using indirect assumptions, such as inter-eye symmetry^6^ or test–retest consistency^7^, because the true latency is unknown. This limitation motivates the use of synthetic PLR data with known ground-truth latency, enabling direct and quantitative comparison of latency detection methods under controlled conditions.

## Methods

### Model implementation

For the purpose of generating synthetic PLR signals under varying conditions (e.g., sampling rate, noise level, and inter-individual variability), a dynamic model that is both computationally efficient and physiologically plausible is required. The biomechanical model by Usui and Hirata^24^ was deemed too complex for this purpose, consistent with the assessment by Fan and Yao^25^. The model by Fan and Yao is specifically tailored to short light pulses^22^, while the hybrid deep-learning approach by Zandi and Khanh^28^ offers limited interpretability and flexibility for systematic parameter variation. Therefore, the model proposed by Pamplona and Oliveira^27^ was selected as the basis for synthetic data generation.

The governing differential equation of t

1 $$\begin{aligned} \frac{dM}{dD} \frac{dD}{dt} + 2.3026 \operatorname {atanh} \left( \frac{D - 4.9}{3} \right) = 5.2 - 0.45 \ln \left[ \frac{\phi (t-\tau )}{\tilde{\phi }} \right] , \end{aligned}$$

with

2 $$\begin{aligned} M(D) = \operatorname {atanh} \left( \frac{D-4.9}{3} \right) , \end{aligned}$$

where *D* is the pupil diameter in mm, *ϕ (t)* the stimulus intensity in lumens at time *t*, $\tilde{\phi }$ is the light level threshold below which there is no longer any pupil response, and *τ * the latency in milliseconds. The function *M*(*D*) models the elastic response of the iris as a function of pupil diameter. This equation combines a dynamic first-order differential system with the equilibrium-state model of Moon and Spencer^26^, ensuring realistic steady-state pupil diameters across varying luminances.

Pamplona and Oliveira account for the asymmetry between pupil constriction and re-dilation, with constriction occurring approximately three times faster than re-dilation^1,29^. This asymmetry is implemented via different integration step sizes $\delta t_c$ and $\delta t_d$ respectively:

3 $$\begin{aligned} \delta t_c = \frac{T_i - T_{i+1}}{S}, \quad \delta t_d = \frac{T_i - T_{i+1}}{3S}, \end{aligned}$$

where $T_i$ and $T_{i+1}$ denote consecutive simulation time steps in milliseconds. The parameter *S* is an individual-specific constant that governs the velocity of pupil constriction and dilation.

**Fig. 1:**
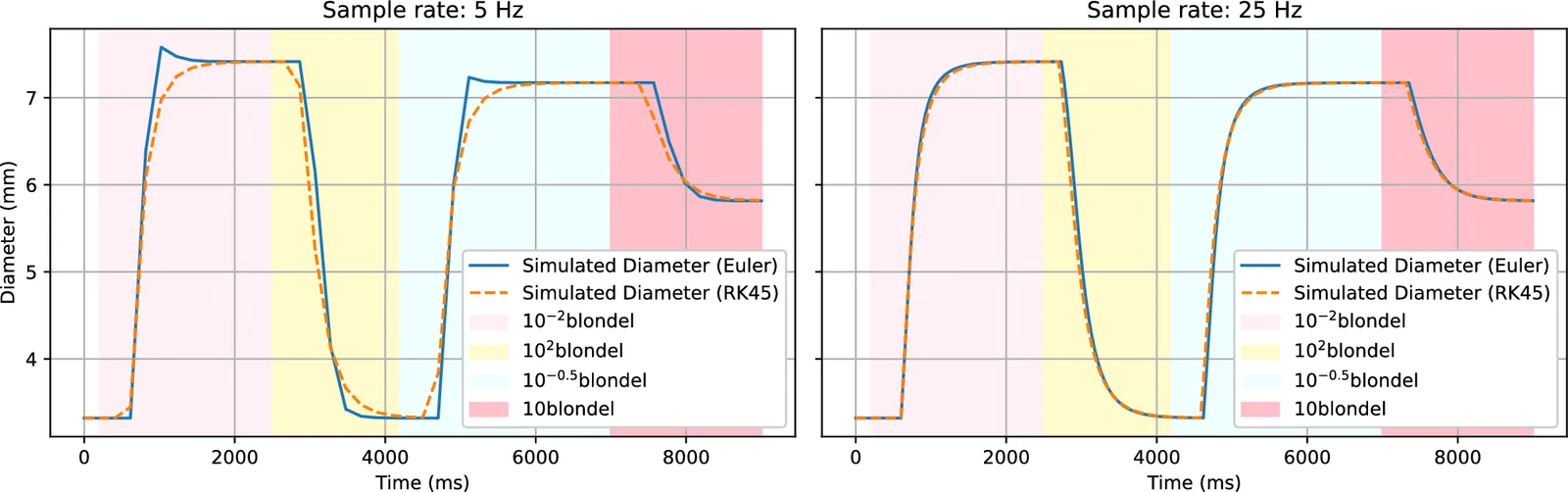
Comparison of the results of our solver (solid line) with the explicit Runge-Kutta method of order 5(4) from SciPy (dotted line). The same illumination levels as those used by Pamplona and Oliveira ($10^2$, $10^2$, $10^{-0.5}$, and 10 blondels) were chosen for this simulation. On the left side, the simulation was performed with a sample rate of 5 Hz, and on the right side with a sample rate of 25 Hz.

**Fig. 2:**
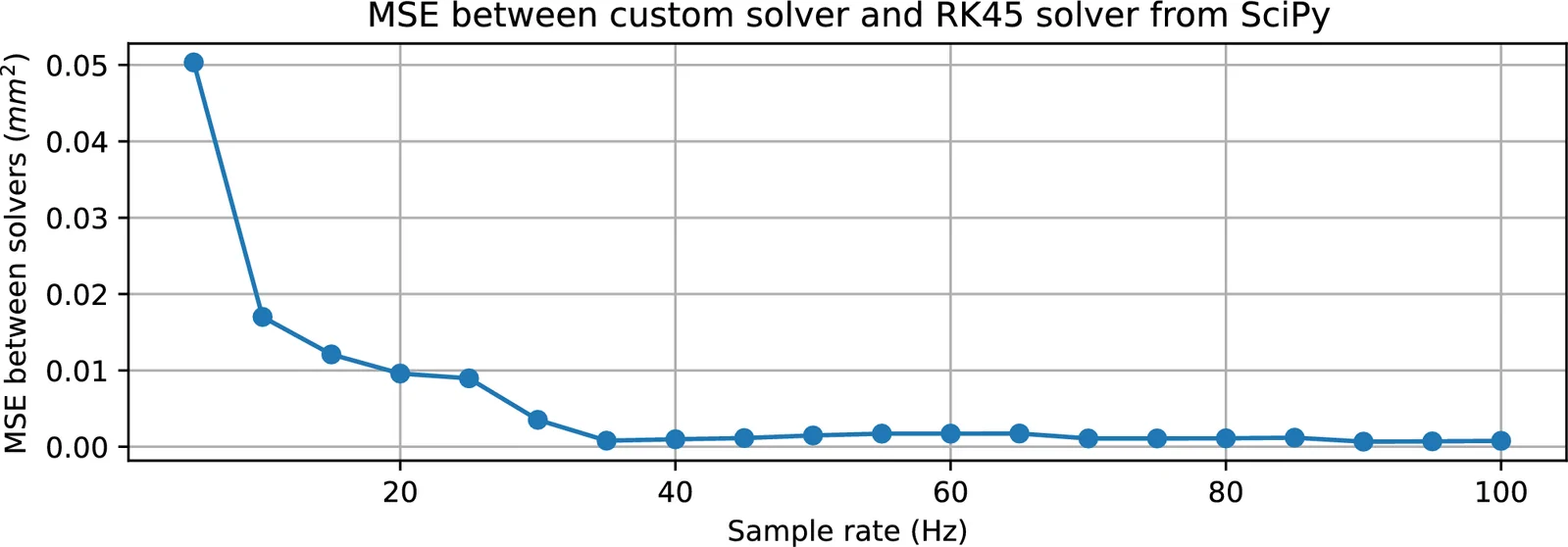
MSE between our custom solver, which uses the Euler method, and an RK45 solver from SciPy. The MSE is evaluated over sample rates between 5 and 100 over a step size of 5.

We implemented a custom Euler-based solver to handle these adaptive steps^30^. While Euler integration is less accurate than higher-order methods^31^, comparison with SciPy’s^32^ explicit Runge–Kutta 5(4) solver (RK45) confirmed acceptable accuracy, with a mean squared error (MSE) below $0.01~\mathrm {mm^2}$ for sampling rates above 25 Hz (Figs. 1, 2).

A known limitation of the Pamplona and Oliveira model is its handling of short light pulses, as previously noted by Fan and Yao^25^. The issue originates from the delayed stimulus term $\phi (t-\tau )$ during numerical integration. The latency *τ * is computed according to Stark’s empirical model^33^:

4 $$\begin{aligned} \tau (R, L_{fL}) = 253 - 14 \ln (L_{fL}) + 70R - 29 R \ln (L_{fL}), \end{aligned}$$

where $L_{fL}$ denotes luminance in foot-Lamberts and *R* the stimulation frequency in Hz.

**Fig. 3:**
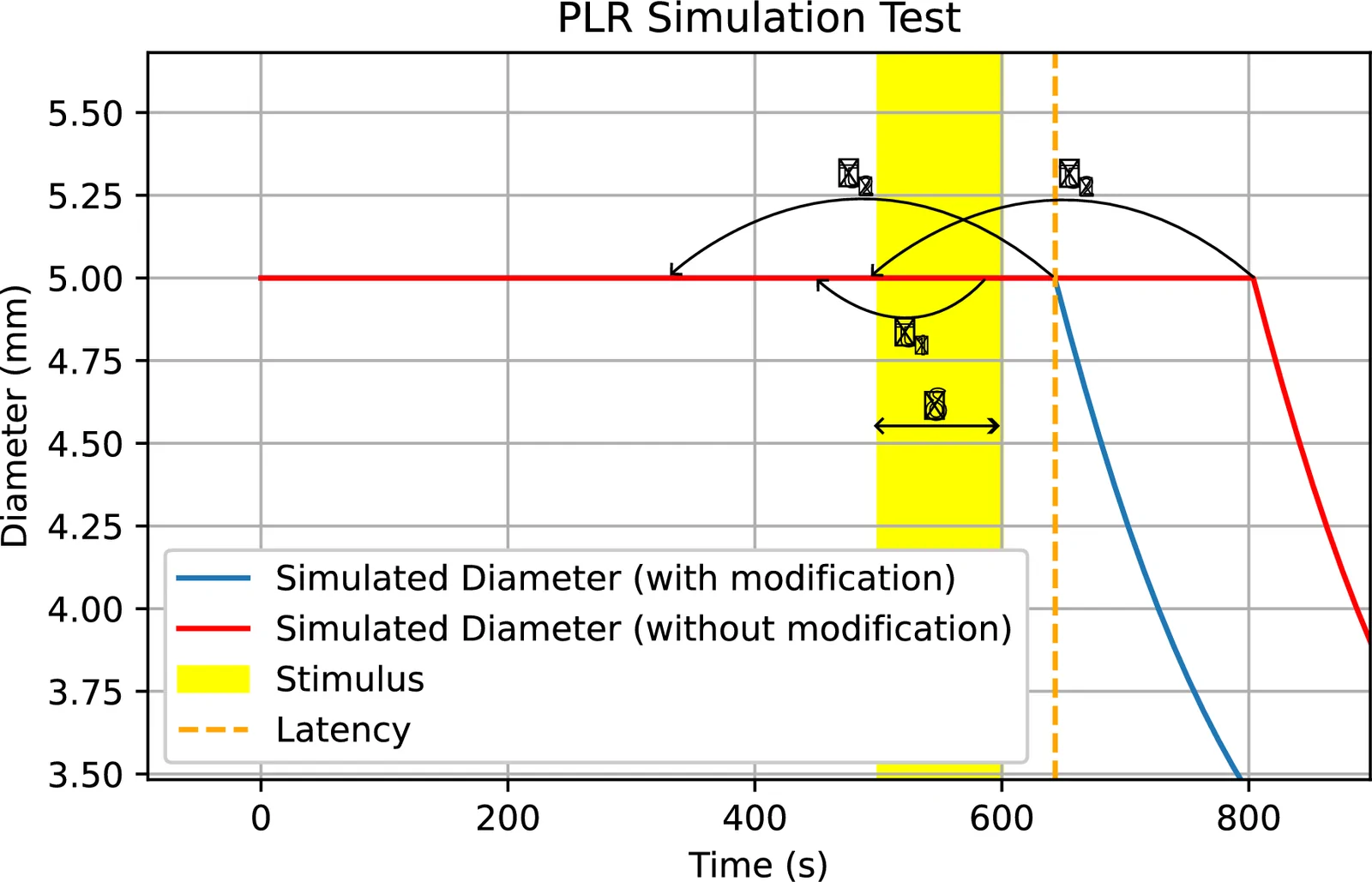
Illustration of default latency handling for a short light pulse and the effect of the proposed modification. A brief stimulus of duration *δ * (yellow region) induces a short latency $\tau _1$ during the pulse and a longer latency $\tau _2$ before and after the pulse. The arrows $\tau _1$ and $\tau _2$ indicate that at time t, $\phi (t-\tau )$ acts on the pupil. The dashed orange line indicates the model-predicted onset of the pupil response according to Eq. (4). Without modification (red curve), the response is delayed because the longer latency $\tau _2$ is active during numerical integration, causing the delayed stimulus evaluation to reference pre-stimulus luminance values. With the proposed modification (blue curve), the response onset aligns with the predicted latency $\tau _1$, resulting in a single continuous pupil response.

The problem can be illustrated by considering a very short light pulse with a duration of *δ * as shown in Fig. 3. According to Stark’s formula, there are then two latencies for the entire process. A short latency $\tau _1$ during the light pulse and a longer latency $\tau _2$ before and after the light pulse. Since the light intensity for the light pulse is known during simulation, Eq. (4) can be used to calculate when the pupil response should begin (dashed orange line). However, since $\tau _2$ is already active again at this time step during numerical integration, a value is calculated for $\phi (t-\tau )$ that lies before the onset of the light pulse. As a result, the pupil’s response may be delayed or even fragmented whenever $\delta < \tau _2$.

To address this issue, the delayed stimulus evaluation in Eq. (1) was modified to ensure that short light pulses elicit a single, continuous response. The model maintains an active latency $\tau _\textrm{active}$ and the time $t_\textrm{active}$ at which it became effective. When a stimulus change occurs, shorter latencies are adopted immediately, while longer latencies are deferred until the preceding shorter latency has elapsed.

Formally, the delayed stimulus is defined as

5 $$\begin{aligned} \phi _\textrm{delayed}(t) = {\left\{ \begin{array}{ll} \phi _\textrm{hold}, & t - \tau _\textrm{active} < t_\textrm{active}, \\ \phi (t - \tau _\textrm{active}), & t - \tau _\textrm{active} \ge t_\textrm{active}, \end{array}\right. } \end{aligned}$$

where $\phi _\textrm{hold}$ denotes the stimulus value immediately before the change that activated the current latency. This modification ensures that brief light impulses with durations shorter than the calculated latency produce a physiologically plausible, uninterrupted pupil response.

Inter-individual variability is modeled following Pamplona and Oliveira by scaling the simulated pupil diameter *D* between subject-specific lower and upper bounds:

6 $$\begin{aligned} D_{\textrm{final}} = C_{bD}(D) + \bigl (C_{tD}(D) - C_{bD}(D)\bigr ) r_I, \end{aligned}$$

where $r_I \in [0,1]$ denotes an individual index. The functions $C_{tD}(D)$ and $C_{bD}(D)$ define the upper and lower envelopes of pupil diameter as a function of luminance, derived from the experimental data of Moon and Spencer^26^. Since the original fifth-degree polynomials reported by Pamplona and Oliveira did not yield realistic values in our simulations, new polynomial approximations were fitted to the mean pupil diameter curve obtained from Moon and Spencer’s data:

7 $$\begin{aligned} C_{tD}(D) = -0.015 D^5 + 0.390 D^4 - 3.913 D^3 + 18.452 D^2 - 38.770 D + 31.368, \end{aligned}$$

8 $$\begin{aligned} C_{bD}(D) = -0.022 D^5 + 0.537 D^4 - 4.935 D^3 + 21.440 D^2 - 43.234 D + 34.305. \end{aligned}$$

The parameter *D* is the pupil diameter calculated using equation (1).

### Data generation

Hippus, the small involuntary oscillations of pupil diameter, is introduced in our simulations to reflect natural variability. While Pamplona and Oliveira^27^ modeled hippus as fluctuations in stimulus brightness, we add it retrospectively to the pupil diameter in order to isolate the response to illumination. The oscillations are implemented as a sinusoid with a frequency between 0.05 Hz and 0.3 Hz^34^ with an amplitude of 0.2 mm^35^.

Synthetic PLR traces were generated parametrically. For a given set of generation parameters—sampling rate, noise level, and stimulus intensity–a population of pupil responses was simulated. Each population consisted of 1000 independent traces to ensure statistically representative error estimates. The ground-truth latency for every trace was calculated using Eq. (4) and used for subsequent evaluation.

Stimulus intensity was controlled indirectly by specifying an average baseline diameter and an average minimum diameter reached after the light pulse; the corresponding stimulus intensity was then determined by the model. Low-intensity stimuli were represented by baseline and minimum diameters of 5 mm and 4 mm, respectively, medium-intensity stimuli used 6 mm and 3.5 mm, while high-intensity stimuli used 7 mm and 3 mm. Inter-individual variability was introduced using the individual index $r_I$ (Eq. 6), which adjusts the final pupil diameters and ensures that the actual response deviates from these representative values. A single value of $r_I$ was drawn uniformly at random for each trace and held constant throughout that trace, so that it represents a fixed individual characteristic rather than a source of within-trace fluctuation.

The stimulus luminances corresponding to these diameter conditions were determined by the model. Following Pamplona and Oliveira, the underlying Moon and Spencer model assumes a large, uniformly illuminated white screen acting as a perfect Lambertian diffuser^26,27^, and expresses stimulus intensity in blondels, where one blondel equals $10^{-6}$ lumens/mm$^2$. For such a screen, one blondel corresponds to a luminance of $1/\pi \approx 0.318$ cd/m$^2$^36^, which we use to report the stimulus strengths below. For the low-intensity condition, the model-equivalent luminances were 13.5 cd/m$^2$ (baseline) and 80.3 cd/m$^2$ (stimulus). For the medium-intensity condition, they were 2.2 cd/m$^2$ (baseline) and 226 cd/m$^2$ (stimulus). For the high-intensity condition, they were 0.19 cd/m$^2$ (baseline) and 788 cd/m$^2$ (stimulus). Retinal illuminance is reported in trolands (Td), defined as the product of stimulus luminance in cd/m$^2$ and pupil area in mm$^2$^36^. Computed at response onset from the baseline pupil area, before the pupil constricts, it is approximately $1.6\times 10^{3}$ Td (low-intensity), $6.4\times 10^{3}$ Td (medium-intensity), and $3.0\times 10^{4}$ Td (high-intensity). Since Moon and Spencer^26^ did not report the spectral composition of the light source in the experiments underlying the model, the model contains no spectral information, except that it applies to white light.

The stimulus duration was fixed at 200 ms for all simulations. Sampling rate and noise level were treated as configurable parameters and varied systematically in the experiments described in the next section.

The underlying delayed differential equation was solved using a fixed internal integration step size of 1 ms for all simulations. The pupil diameter time series obtained from this high-resolution integration was subsequently sampled at the target sampling rates used in the experiments. This two-stage procedure reduces discretization-induced shifts of the response onset that may arise when integrating directly at low sampling rates, and ensures consistent temporal accuracy across all experimental conditions, including low-rate configurations.

### Empirical validation

For qualitative validation of the benchmark with real measurements, five PLR recordings were acquired from a single subject using a custom-built laboratory pupillometer^37^. This experiment serves as a plausibility check to assess whether the benchmark findings transfer to empirical data. Ethics approval was obtained from the institutional research ethics committee of Leibniz University Hannover (ID: EV LUH 13/2025). All methods were performed in accordance with relevant guidelines and regulations, including the Declaration of Helsinki. Written informed consent was obtained from all participants.

The stimulus was a diffused 5 mm white LED presented for 1 s at 75 lx measured at the eye, and recorded at 30 fps. As no datasheet was available, the LED spectrum was measured directly and showed the broadband profile of a phosphor-converted white LED, with a blue peak near 450 nm and a phosphor peak near 520 nm spanning approximately 425–695 nm, so no single stimulus wavelength is defined.

## Experiments

**Fig. 4:**
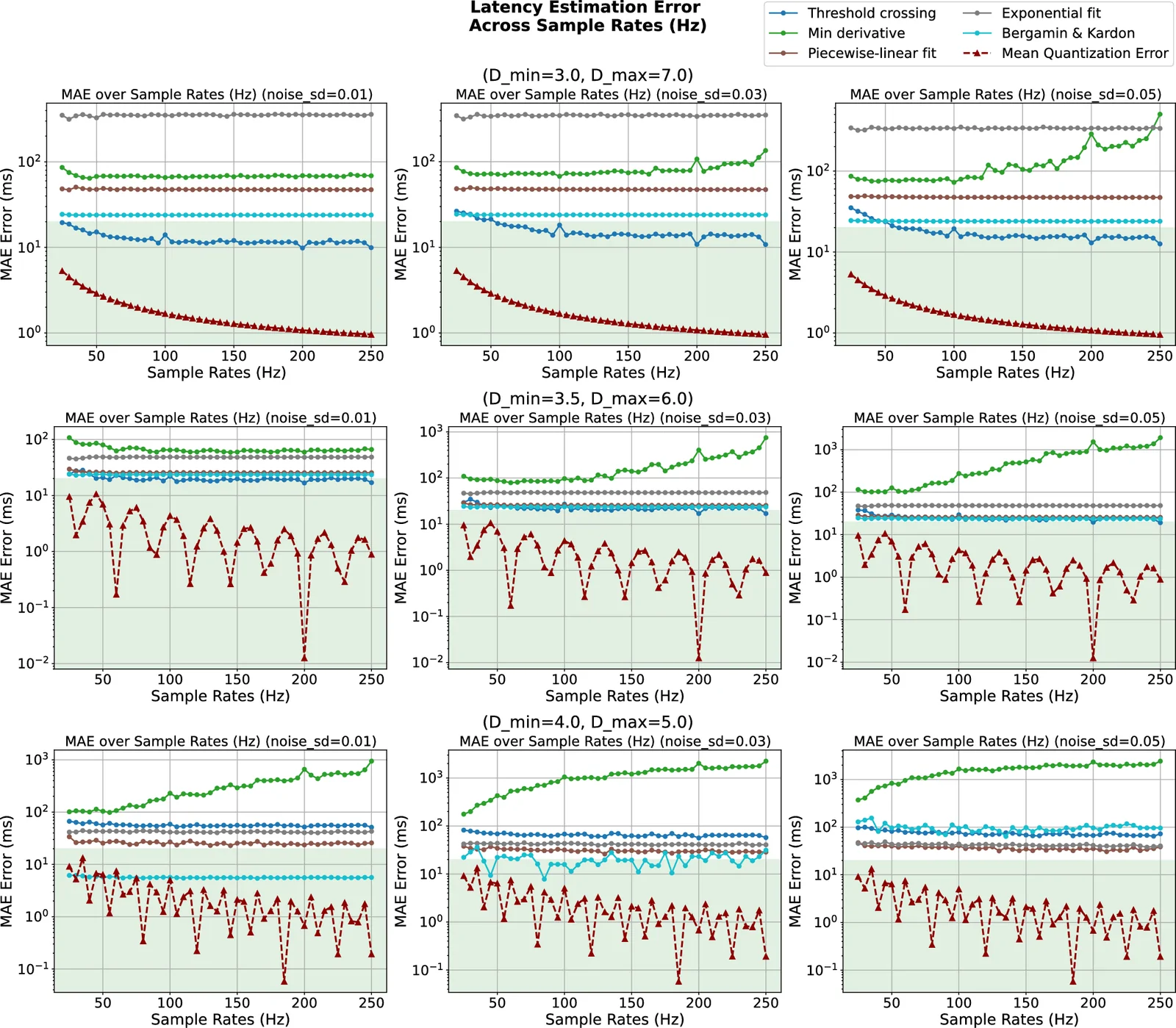
Mean absolute error (MAE) of all evaluated latency estimation methods as a function of sampling rate. Columns correspond to increasing noise levels (*σ * = 0.01, 0.03, 0.05 mm, left to right). The top row shows results for high-stimulus conditions, the middle row for medium-stimulus conditions, while the bottom row shows results for low-stimulus conditions. The x-axis spans sampling rates from 25 Hz to 250 Hz in steps of 5 Hz. The green area indicates MAE below 20 ms. The *mean quantization error* curve shows the sampling-imposed lower bound on the MAE and is included for reference, not as an estimation method..

**Fig. 5:**
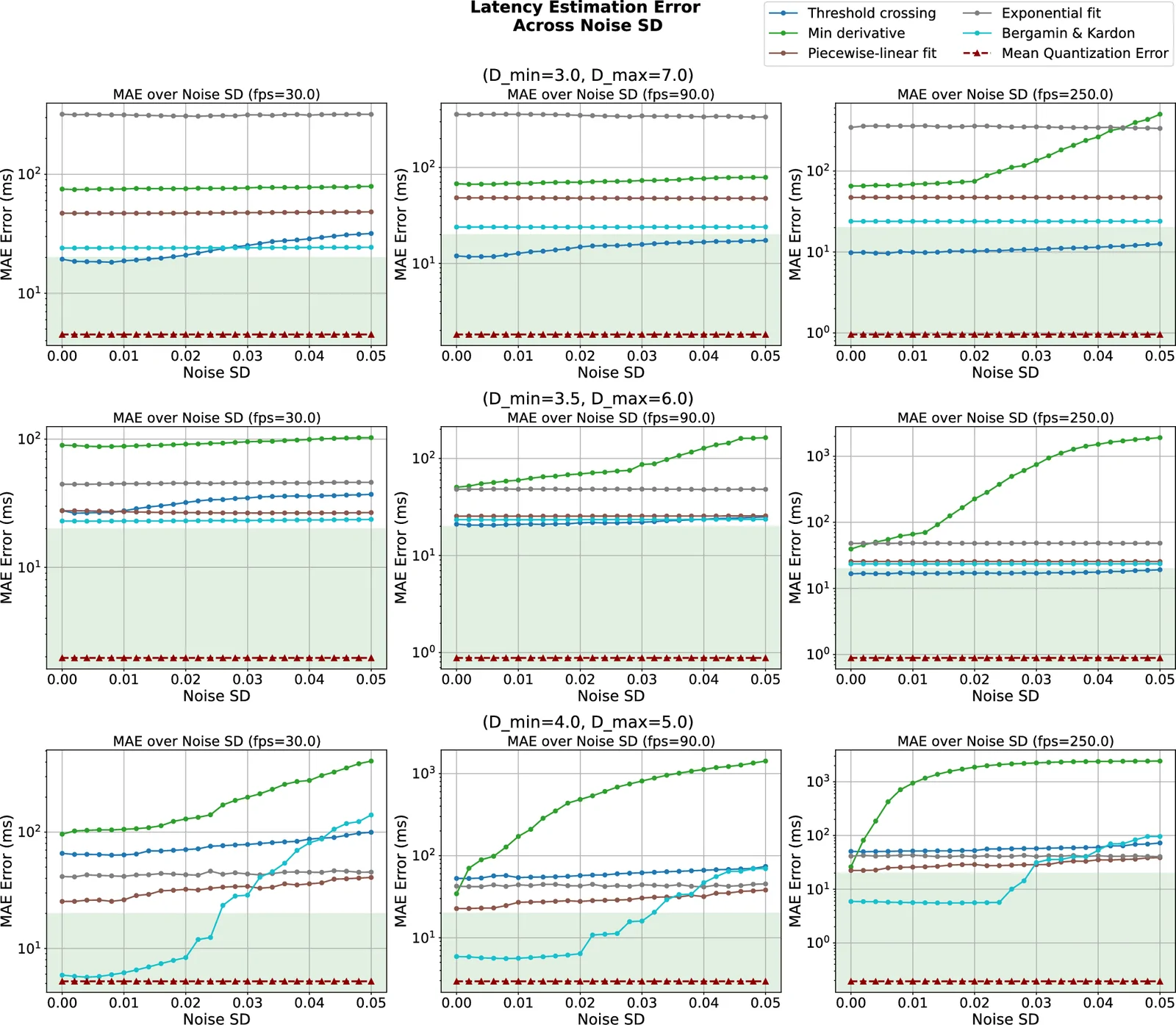
Mean absolute error (MAE) of all evaluated latency estimation methods as a function of noise level. Columns correspond to different sampling rates (30 Hz, 90 Hz, and 250 Hz, left to right). The top row shows results for high-stimulus conditions, the middle row for medium-stimulus conditions, while the bottom row shows results for low-stimulus conditions. Gaussian noise standard deviation ranges from *σ * = 0 to 0.05 mm in steps of 0.002 mm. The green area indicates MAE below 20 ms. The *Mean Quantization Error* curve shows the sampling-imposed lower bound on the MAE and is included for reference, not as an estimation method..

Five latency estimation methods were evaluated in our experiments. The first method is the intersection approach proposed by Jendritza et al.^7^, based on the intersection of a linear fit through the baseline with a linear fit through the contraction phase (shown as *Piecewise-linear fit* in the legends of Figs. 4 and 5). The second method corresponds to Bos’s^15^ preferred approach for high sampling rates, which identifies velocity deflections from zero (*Min derivative*), while the third method evaluates Bos’s approach for low sampling rates, where curve fitting is recommended (*Exponential fit*). The fourth method is that of Bergamin and Kardon^6^ (*Bergamin & Kardon*), and a simple threshold method (*Threshold crossing*) was included as a baseline.

For the simple threshold method, latency was estimated as the time when the pupil diameter first fell below three times the standard deviation of the baseline. For linear-fit-based methods, SciPy’s least-squares solver^32^ was used. To avoid heuristic choices regarding the number of points for fitting, a piecewise linear model was implemented in which both lines and the breakpoint are estimated simultaneously under a continuity constraint. This concept was proposed by Bos for his second-order curve-fitting method, which models the pupil response using two exponential functions^15^, and was here extended to the linear intersection method. For the second-order curve-fitting method, the pupil diameter is described by a linear segment before the onset *T* and by a difference of two exponentials afterwards:

9 $$\begin{aligned} D(t) = {\left\{ \begin{array}{ll} a_0 + b_0\,(t - T), & t < T,\\ a_1 + a_2\,e^{-b_2 (t - T)} - a_3\,e^{-b_3 (t - T)}, & t \ge T, \end{array}\right. } \end{aligned}$$

with the continuity constraints $a_0 = a_1 + a_2 - a_3$ and $b_0 = -a_2 b_2 + a_3 b_3$, which keep the curve and its first derivative continuous at the breakpoint *T*, taken as the estimated latency^15^. For Bos’s high-sample-rate method, the maximum negative velocity was used instead of a zero-crossing due to noise-induced fluctuations.

For Bergamin and Kardon’s method^6^, we followed the procedure described in the original publication. The pupil signal was first smoothed using a Savitzky-Golay filter (window size of 5 time steps, second-order polynomial), then interpolated to 300 Hz. The first derivative was calculated and further smoothed with a Gaussian filter (*σ = 25*) to obtain velocity, followed by a second derivative to compute acceleration. Latency was defined as the point of maximum negative acceleration^6^.

The performance of latency estimation methods was evaluated using synthetic pupillometry data with known ground-truth latency. For all experiments, the MAE between the estimated and true latency was used as the evaluation metric. Reported MAE values represent averages over populations of 1000 independently generated PLR traces per experimental configuration.

Two complementary experiments were conducted. In the first experiment (Fig. 4), the sampling rate was varied, while the measurement noise was held constant. Sampling rates between 25 Hz and 250 Hz were evaluated, covering the range of modern image-based pupillometers. Lower sampling rates were not considered, as the numerical solver accuracy deteriorates below this range, while higher rates were excluded due to limited relevance for camera-based systems and increased computational cost. The noise standard deviation was fixed at 0.01, 0.03 and 0.05 to illustrate interactions between noise and temporal resolution. The mean value of the noise was always set to 0 for all configurations and all experiments. For each sampling rate, latency estimation methods were applied, and the MAE was computed. In the corresponding plots, the sampling rate is shown on the x-axis, MAE on the y-axis, and each curve represents a different latency estimation method.

In the second experiment (Fig. 5), the noise level was varied while the sampling rate was fixed. Gaussian noise is assumed for the experiments, but can easily be replaced by other noise behaviors. Standard deviations between 0 mm and 0.05 mm were applied to simulate measurement inaccuracies. Steinhauer et al.^38^ report pupil diameter measurement errors in the range of approximately 0.01–0.05 mm for video-based pupillometry systems. Although Gaussian noise is unbounded and individual samples may exceed ±0.05 mm, the selected noise levels reflect the scale of reported pupil diameter measurement errors. Rare larger deviations were tolerated to avoid artificial truncation of the noise distribution and to assess robustness under slightly adverse conditions. The sampling rate was fixed at 30 Hz and 90 Hz, reflecting commonly used and high-frequency video-based pupillometry systems, respectively. Additional results at 250 Hz are reported as a reference to illustrate interactions between noise and temporal resolution. As before, MAE values were computed over populations of 1000 traces for each noise level. In these plots, the noise standard deviation is shown on the x-axis, MAE on the y-axis, and curves correspond to latency estimation methods.

All experiments were performed separately for low-, medium-, and high-intensity stimuli, as defined in the “Data generation” subsection, to assess the robustness of latency estimation methods under varying constriction amplitudes. Ground-truth latency was identical across methods within each condition.

In Figs. 4 and 5, MAE values below 20 ms are highlighted in green. This threshold is based on Bos’ statement that latency differences of more than 20 ms are considered unacceptable^15^, as discussed in the “Introduction”. It should be understood as a pragmatic reference value derived from a single methodological recommendation rather than a validated clinical requirement. Its practical relevance is nonetheless underlined by the small effect sizes reported clinically^2^.

## Results

In many tested conditions, the method proposed by Bergamin and Kardon (*Bergamin & Kardon*) exhibited promising results. Interestingly, its performance decreased at higher noise levels under low-intensity stimulus conditions, despite being the only method to explicitly incorporate filtering to mitigate noise. This degradation, however, was slightly compensated by an increased sampling rate, indicating that higher temporal resolution improves robustness. One possible explanation is that the Gaussian noise used in our simulations, while commonly used to approximate measurement noise, may not fully capture all characteristics of noise present in real pupillometry recordings. As a result, the filtering strategy employed by Bergamin and Kardon may not interact with the simulated noise in the same way as with naturally occurring noise. The method using the minimum value of the first derivative (*Min derivative*) was also susceptible to noise. And although this approach was used as representative of the method recommended by Bos for high sampling rates, performance does not improve at higher sampling rates and even deteriorates with higher sampling rates in the low-stimulus experiments or with increasing noise. The method recommended by Bos for lower sampling rates, a second-order curve fit (*Exponential fit*), also does not appear to be affected by the sampling rate and exhibited the poorest performance under high-intensity stimulus conditions. In high- and medium-intensity stimulus conditions, the simple threshold method (*Threshold crossing*) exhibited competitive performance. However, its accuracy decreased under low-intensity stimulus conditions, indicating limited robustness to small pupil constrictions. The piecewise linear fit method (*Piecewise-linear fit*) demonstrated consistent, average performance across all sampling rates and noise levels, showing little sensitivity to parameter variations. This stability suggests that the approach may be well suited for scenarios with unknown hardware specifications or as a straightforward initial method for latency estimation.

Surprisingly, the predefined quality criterion of an MAE of less than 20 ms is often not achieved. This result is particularly interesting because the difference between the mean values of the control group and subjects with concussion in the study by Capó-Aponte et al.^2^ was <30 ms, which is not much higher than the selected threshold value. This also shows how important good spatial and temporal resolution is in pupillometry. If both are not given, we advise against regarding latency as a meaningful biomarker.

To indicate the variability behind the mean MAE curves, Table 1 reports, for each method, the MAE and the standard deviation of the absolute error averaged across all conditions of each experiment. The standard deviations are large, frequently exceeding the corresponding mean, which reflects high variability across the 1000 traces and substantial overlap between the methods’ error distributions. Rather than undermining the benchmark, this variability reinforces one of its findings, namely that none of the tested methods was accurate enough across every configuration to be considered clinically reliable, and the large spread of errors underlines how strongly the estimated latency depends on the recording conditions. This finding is also reported in the clinical literature, where the constriction latency is an inconsistent marker of mild traumatic brain injury, reported as significantly prolonged in some cohorts but statistically indistinguishable from controls in others^4^. The Bergamin and Kardon method attained the lowest mean error but also a higher variability than the threshold-crossing and piecewise-linear methods. A possible cause is its occasional large errors under high noise and low-intensity stimuli. The threshold-crossing and piecewise-linear methods were the most consistent, whereas the exponential fit and, in particular, the min-derivative method showed both high error and high variability.

**Table 1:** Mean absolute error (MAE) and standard deviation (SD) of the absolute error for each method, averaged across all conditions of each experiment. The large SDs, often exceeding the mean, indicate high variability across the 1000 traces per configuration and substantial overlap between methods.

Method	Sampling-rate exp.		Noise exp.	
	MAE (ms)	SD (ms)	MAE (ms)	SD (ms)
Bergamin & Kardon	29.6	99.7	26.1	77.9
Threshold crossing	34.4	54.1	35.1	52.6
Piecewise-linear fit	34.8	63.4	34.7	63.1
Exponential fit	145.3	71.1	142.6	72.2
Min derivative	458.1	616.4	421.1	507.4

We further compared the benchmark against the empirical recordings described in the “Empirical validation” subsection. The simulation frame rate and stimulus duration was matched to that of the pupillometer, and the noise level of the synthetic data was adjusted by visual comparison to approximate the measurement noise observed in the empirical recordings. The minimum and maximum pupil diameters in the simulation were set to 4 mm and 6 mm, respectively, to roughly correspond to the diameter range observed in the recorded data.

**Fig. 6:**
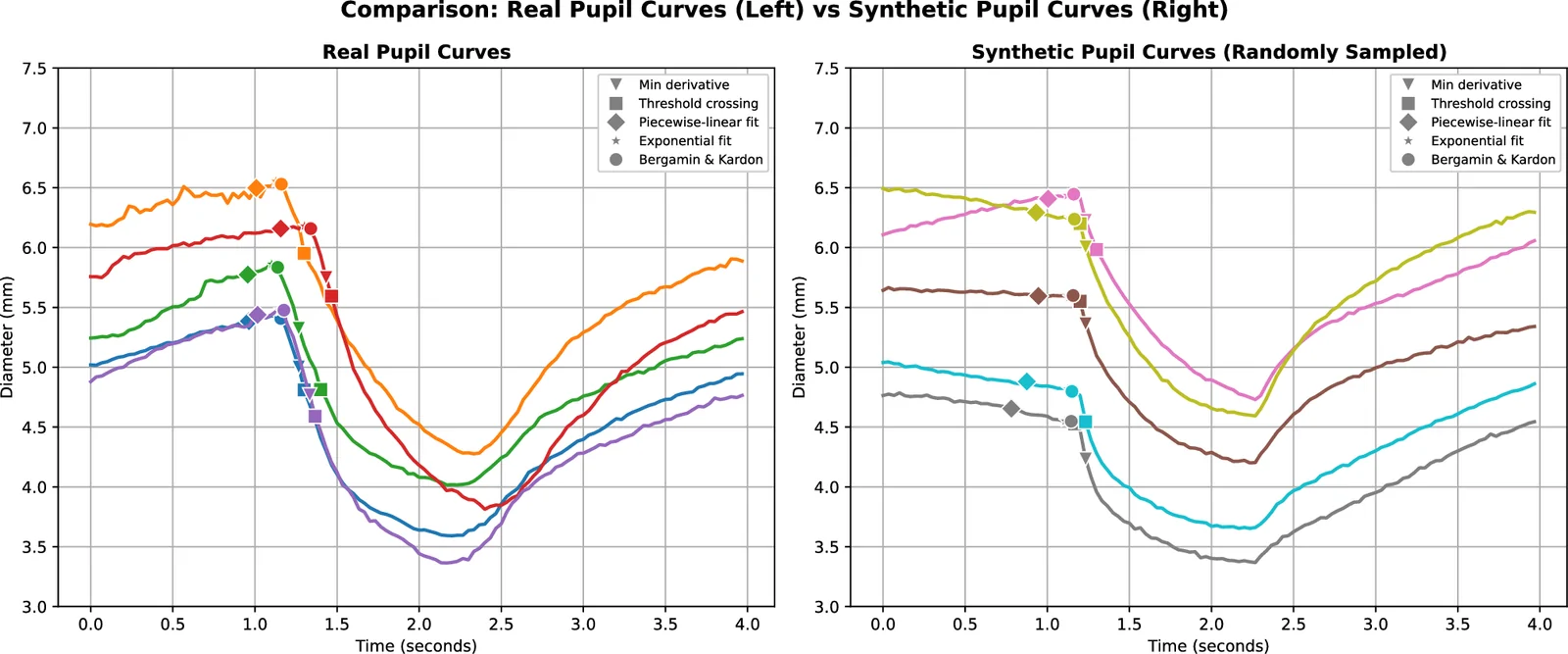
Comparison between empirical and simulated PLR recordings. Left: five PLR curves recorded using a custom-built laboratory pupillometer. Right: five randomly selected simulated pupillograms generated with matched sampling rate, noise level, and pupil diameter range (4–6 mm). Markers indicate the latency estimates of all five evaluated methods, as shown in the legend..

At the matched configuration, the benchmark ranked the methods, from lowest to highest MAE, as Bergamin and Kardon (28.8 ms), min derivative (39.1 ms), threshold crossing (50.6 ms), exponential fit (60.3 ms), and piecewise-linear fit (232.5 ms). Figure 6 compares the five empirically recorded pupillograms with five randomly selected simulated pupillograms generated under the matched conditions. On the real recordings, the piecewise-linear fit marked the onset too early, the min-derivative and threshold methods marked it too late, and the Bergamin and Kardon and exponential fit methods fell close to the visible onset. On the synthetic curves the same directions are visible, but the late marking of the min-derivative and threshold methods is less pronounced. That the exponential fit lies close to the onset in both the real and the synthetic samples shown here, despite its higher benchmark MAE, may reflect the limited significance of only five samples.

While the simulated traces exhibit a sharper transition between constriction and redilation than the empirical recordings, these differences do not affect latency estimation and are therefore not critical for the scope of this study.

## Discussion

Our evaluation of latency estimation methods on synthetic pupillometry data highlights differences in robustness and accuracy. The method by Bergamin and Kardon achieved the most promising results, but its performance declined under high noise with low-intensity stimuli, an effect that could be mitigated by higher sampling rates, indicating that temporal resolution remains a critical factor for accurate latency estimation. Because it achieved a consistently low error in most tested conditions, except under high noise in the low-stimulus experiments, we recommend the Bergamin and Kardon method as the default choice for latency estimation whenever the measurement hardware is known and can be controlled.

The piecewise linear fit method exhibited consistent, average performance across all tested configurations, indicating that it may be well suited for applications with unknown hardware specifications, such as smartphone-based pupillometry. However, it should be noted that the method of implementation has impact on the prediction. Unlike Jendritza’s approach^7^, in which optimal fitting windows were carefully determined, our implementation uses only one window, from the start of the sequence to the point at which the minimum pupil diameter was reached. As mentioned in the “Experiments” section, our approach relies on a continuity constraint to fit both lines simultaneously. In the high-stimulus setup, the line fitted to the constriction phase is often influenced by its later part, where the constriction has already slowed down, which shifts the estimated breakpoint and increases the latency error. Optimizing the fitting window is, however, not straightforward, since the most suitable window may itself differ between stimulus strengths. Moreover, shorter windows are more strongly affected by physiological fluctuations such as hippus or baseline shifts, so that narrowing the window may introduce other sources of error.

A further obstacle to comparability across studies is that most pupillometers are commercial devices whose latency estimation algorithm is not disclosed by the manufacturer. Because the choice of algorithm can substantially affect the estimated latency, as shown in this work, we argue that manufacturers should clearly state which algorithm their devices use. As an alternative, we would like to encourage researchers to conduct research in the field of open-source pupillometry, as described in^37^. Laboratory prototypes with controlled parameters are a further option, but because such custom setups differ from one laboratory to another, they can themselves make direct comparison between studies more difficult.

The methods also differ in computational cost: the threshold and derivative methods run in a single pass, whereas the piecewise-linear and exponential fits iteratively solve a non-linear least-squares problem. This cost is negligible, however, compared to the pupil detection required to obtain the diameter signal, so it is not a limiting factor even on resource-constrained devices.

Limitations of this study include the use of synthetic data, which, while providing ground-truth latency, may not capture all complexities of real physiological responses. Noise was modeled as additive Gaussian, which may differ from real-world measurement noise in pupillometers. Future work should further investigate the transferability of these results to empirical data and explore whether the incorporation of filtering techniques could improve the performance of methods that currently do not employ them, as only the Bergamin and Kardon method explicitly uses filtering.

## Data Availability

The code used in this research is publicly available at: https://github.com/PLR-Analyzer/PLR-Latency-Benchmark A permanently archived version is deposited in the research data repository of Leibniz University Hannover and is available at:394 https://doi.org/10.25835/0ugtwxbm.
